# Chemiluminescent immunoassay technology: what does it change in autoantibody detection?

**DOI:** 10.1007/s13317-017-0097-2

**Published:** 2017-06-24

**Authors:** Luigi Cinquanta, Desré Ethel Fontana, Nicola Bizzaro

**Affiliations:** 1 Autoimmunologia e Allergologia Diagnostica di Laboratorio, UOC di Patologia Clinica, Azienda Ospedaliera Universitaria “Scuola Medica Salernitana”, OORR San Giovanni di Dio e Ruggi d’Aragona, Salerno, Italy; 2 Dipartimento di Medicina di Laboratorio e Istituto di Patologia Clinica, Azienda Sanitaria Universitaria Integrata di Udine, Udine, Italy; 3 Laboratorio di Patologia Clinica, Ospedale San Antonio, Azienda Sanitaria Universitaria Integrata di Udine, Tolmezzo, Italy

**Keywords:** Chemiluminescence, Autoimmune diseases, Autoimmunology laboratory, Automation

## Abstract

Diagnostic technology is rapidly evolving, and over the last decade, substantial progress has been made even for the identification of antibodies, increasingly approaching this type of diagnostic to that of automated clinical chemistry laboratory. In this review, we describe the analytical and diagnostic characteristics of chemiluminescence technology in its strength and in its applicability for a more rapid and accurate diagnosis of autoimmune diseases. The wide dynamic range, greater than that of immunoenzymatic methods, the high sensitivity and specificity of the results expressed in quantitative form, the high degree of automation and the clinical implications related to the reduction in the turnaround time, and the ability to run a large number of antibody tests (even of different isotypes), directed towards large antigenic panels in random access mode, make this technology the most advanced in the clinical laboratory, with enormous repercussions on the workflow and on the autoimmunology laboratory organisation. Further improvements are expected in the coming years with the development of new analytical platforms such as the flow-injection chemiluminescent immunoassay, the two-dimensional resolution for chemiluminescence multiplex immunoassay and the magnetic nanoparticles chemiluminescence immunoassay, which will likely result in additional increases in the clinical efficacy of antibody tests.

## Introduction

Autoantibody determination is crucial for the diagnosis of many autoimmune diseases, both systemic ones—such as systemic lupus erythematosus (SLE), rheumatoid arthritis (RA), Sjögren’s syndrome, systemic sclerosis and antiphospholipid syndrome- and organ-specific ones, such as coeliac disease, autoimmune thyroid diseases, primary biliary cirrhosis and autoimmune hepatitis. In particular, autoantibody determination is included among classification and/or diagnostic criteria for some autoimmune diseases [1–8] and assumes a relevant predictive value in others, as autoantibodies can appear years before clinical manifestation of disease [9–17].

Numerous analytical methods have been proposed for autoantibody detection. Of these, immunoassay has undergone important and radical changes in recent years due to continuous technological development which has been spurred on by an increased demand for services analogous to that already occurring in other sectors of modern laboratory diagnostics. Fundamentally, immunoassay evolution corresponds to the evolution of immunoassay labelling technology. Radioimmunoassay (RIA) was the first immunoassay to be developed and could be considered the forefather of the modern immunoassay [18]. This revolutionary development of the first RIA for insulin in the late 1950s earned Solomon Berson and Rosalyn Yalow the Nobel Prize. The two researchers chose deliberately not to patent the analytical procedure, and this decision contributed in no small way to the enormous development of immunoassay techniques in the following years. Roger P. Ekins became the architect of vital theoretical and application contributions in subsequent decades. He, along with other researchers, affirms these techniques to their present-day extent [19–23]. The latest non-isotopic label applied to immunochemical techniques is also the earliest, occurring widely in the animal kingdom (as in the firefly) as a light emission system: bioluminescence.

## Chemiluminescence technology

Chemiluminescent immunoassay (CLIA) is an immunoassay technique where the label, i.e. the true “indicator” of the analytic reaction, is a luminescent molecule. In general, luminescence is the emission of visible or near-visible (*λ* = 300–800 nm) radiation which is generated when an electron transitions from an excited state to ground state. The resultant potential energy in the atom gets released in the form of light. In spectrophotometry, luminescence has an advantage over absorbance in that the former is an absolute measure whereas the latter is relative. We make reference to chemiluminescence, because the type of luminescence applied to immunoassay techniques generally identifies exergonic chemical reactions as the most suitable energy source for producing the electronic excited state.

The heterogenous method is the more widely used chemiluminescent assay. Chemiluminescent methods can be direct—using luminophore markers—or indirect—using enzyme markers. Either method may be competitive or non-competitive.

In direct chemiluminescent methods, the luminophore markers used are acridinium and ruthenium esters, while the enzymatic markers used in indirect methods are alkaline phosphatase with adamantyl 1, 2-dioxetane aryl phosphate (AMPPD) substrate and horseradish peroxidase with luminol or its derivatives as substrate.

**Fig. 1:**
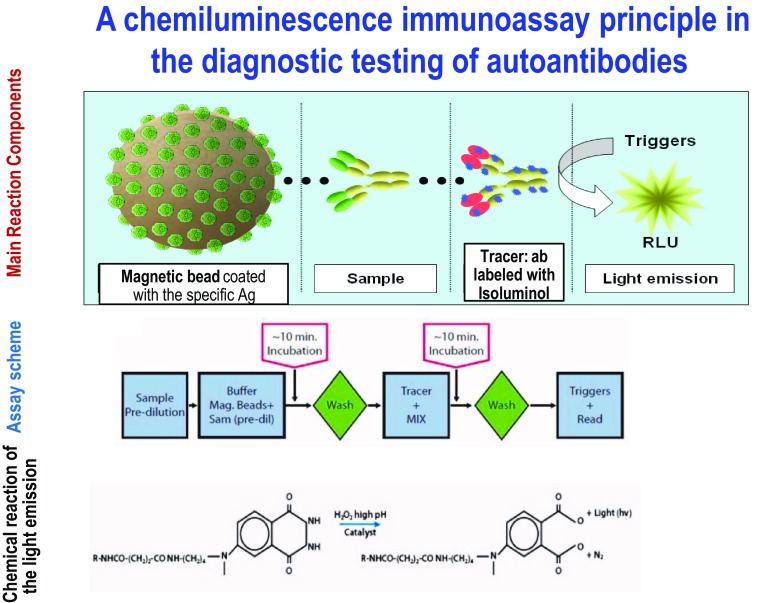
Illustration of BIO-FLASH system, a widely used CLIA in the field of autoimmunity

Synthesising molecules such as AMPPD and isoluminol base molecule derivatives are more stable compared to other luminescent markers and result in light emission with a characteristically elevated quantum yield. Activation of these substrates requires chemical or enzymatic reactions associated with the immunological reaction. For example, the use of luminol and derivatives of isoluminol as chemiluminescent labels depends on the coupling of the immunoassay with enzymatic reactions catalysed by peroxidase (Fig. 1). The addition of an enhancer (e.g. ferrocyanide, metallic ions) further boosts the electronic activation, ultimately leading to extremely elevated analytic sensitivity (mol^−16^ per litre), certainly superior to those attainable by other immunoassay methods such as RIA, immunoenzymatic (ELISA) and fluoroimmunoenzymatic (FEIA) methods, etc.

Luminous signalling, with its constant kinetic curve, contributed to the development of relatively uncomplicated automatic CLIA instrumentation.

CLIA instruments steadily infiltrated the immunometric assay domain, eventually being used to measure serum concentrations of hormones, drugs, vitamins, tumour markers, infectious disease markers, myocardial damage markers and, finally, autoantibodies. Today, autoantibody detection in immunochemiluminescence can be carried out on instruments specifically dedicated to the autoimmunology laboratory as stand-alone instrumentation or as part of an automated analytical platform.

## Advantages and limitations of CLIA technology for the autoimmune laboratory

The key advantages of chemiluminescent analytical methods reside in the wide dynamic range, high signal intensity, absence of interfering emissions (i.e. high specificity), rapid acquisition of the analytical signal, high stability of reagents and their conjugates, low consumption of reagents, random access, reduced incubation time and full compatibility with immunology assay protocols (homogenous or non-homogenous) [24]. Some limitations are to be considered as well. The disadvantages of CLIA are represented by:

- Limited Ag detection
- High costs
- Limited tests panel
- Closed analytical systems.

Furthermore, for many chemiluminescent (CL) systems, there is a low background level of emission in the absence of analyte. Hence, CL signals in flow systems, increasing proportionally to the analyte concentration, appear as sharp peaks superimposed on a low constant blank signal, measured as viewed by the time window when the mixture of analyte and reagent(s) passes through the detector cell. Due to the small portion of CL emission that is only measured from this time profile, reactions with complex kinetics can give nonlinear plots of response versus analyte concentration.

## Automation and standardisation of the method

The use of automated analytical platforms based on CLIA technology brings about solid-phase immunochemical reactions with significantly shorter execution times (30–40 min) than other types of immunoassay. The absolute standardisation of the most critical analytical stages, pertaining to the high stability of reagents and conjugates, contributes in a substantial way to the duration (several weeks) of the stability of the calibration curve. Highly advanced software manages the analysing instruments and supplies automatic processing of the analytical result, internal quality control management and continuous monitoring of every aspect of the analytical phases. Moreover, it provides univocal recognition of patient and quality control samples and of specific and common reagents. In summary, an operational model characterised by complete control of the analytical process is achieved, enabling the autoimmune laboratory to offer the same level of security to the patient as offered by the clinical chemistry laboratory. Indeed, the same characteristics are also present in other immunoassay analysers based on different technologies as fluorescence enzyme immunoassay (FEIA) and Luminex xMap, a bead-based multiplexed immunoassay system. In addition, this latter analytical is particularly powerful when looking for changes in concentrations of multiple autoantibodies under different conditions.

## New scenarios associated with a wider dynamic range

The wide dynamic range that characterises CLIA methods implies a higher analytical sensitivity and the capacity to accurately detect elevated concentrations of antibodies without the need to dilute the sample. For example, a difference in dynamic interval amplitude (2–3 orders of magnitude) has been shown between CLIA assay and that of ELISA and FEIA for the measurement of anti-transglutaminase antibodies (tTG), which has important implications for clinical management of the patient. Lakos demonstrated that, in approximately 50% of adult patients with coeliac disease confirmed by duodenal biopsy, levels of anti-tTG IgA as measured in CLIA were more than 10 times above the upper limit of normal (ULN). This positivity rate proved distinctly superior to that achieved by testing the same samples using the FEIA method [25]. Therefore, because of its wide dynamic range, utilising CLIA methods for detecting anti-tTG IgA could significantly reduce the need for duodenal biopsy for diagnosing coeliac disease in adults. The European Society for Pediatric Gastroenterology, Hepatology, and Nutrition (ESPGHAN) guidelines for coeliac diagnosis in children already provide for reduced dependence on biopsy [26]; the guidelines suggest a cut-off value of >10 × ULN for identifying paediatric patients who might avoid duodenal biopsy, since laboratory test results in such instances could be considered diagnostic even in isolation.

Furthermore, it was demonstrated in a collaborative international study that a strong correlation exists between the serum concentration of anti-dsDNA, as measured by CLIA instrumentation, and the SLEDAI-2K score currently used for monitoring SLE disease activity [27]. This could be explained by the CLIA method’s wide dynamic range for detecting anti-dsDNA antibodies, unlike that of the ELISA method with which it was compared.

## Analytical sensitivity and predictive value

CLIA technology permits analytical procedures with lower analyte detection limits than other immunoassay methods. In other words, CLIA is able to determine the presence of antibodies at extremely low concentrations (limit of detection = zeptomole 10^−21^ mol). Considering what we know regarding the predictive value of certain autoantibody specificities, this characteristic could be of fundamental importance. Numerous autoimmune diseases are chronic in nature, progress over a time span of years and are characterised by specific autoantibodies the presence of which can precede full-blown disease by many months or many years. Consider, as illustrations, the simultaneous presence of two different autoantibodies against pancreatic islets: this is associated with a 50% increased risk of developing type I diabetes mellitus within 5 years [28]; or the presence of anti-cyclic citrullinated peptide antibodies (anti-CCP): they appear in the serum of patients with RA on average 4–5 years prior to full-blown disease [29, 30]; or the presence of specific circulating autoantibodies (anti-nucleosome, anti-Ro, anti-phospholipids and anti-P ribosomal protein): these appear in patients affected by SLE up to 5 years before clinical manifestation of disease [9, 31, 32]. The possibility to predict autoimmune disease—or, rather, their clinical manifestations—presents the opportunity to screen healthy individuals for autoantibodies using analytical methods with the highest sensitivity. The significance of this prospect appears evident when we consider the importance of promptly identifying life-threatening situations like Addisonian crisis and thyroid storm, and of anticipating treatment and thus preventing clinical manifestations of autoimmune disease in the preclinical stages [33].

## Increased productivity and workflow modifications

Adopting CLIA, or technologies as FEIA and Luminex, for autoantibody detection leads to a significant increase in productivity for the autoimmunology laboratory in order to cope with the ever-expanding array of autoantibodies proposed by the world of scientific research and the continuous increase in test requests from various clinical disciplines resulting from enhanced awareness of autoimmune pathologies. The introduction of automated immunoassay analysers, which can execute 60–70 tests h^−1^, allows for the daily workload represented by the most frequently requested autoantibody tests to be completed in just a few hours, thus determining a radical change in the organisation and operation of the clinical laboratory. These automated immunoassay analysers allow a patient’s multiple randomly selected autoantibody tests to be executed either contemporaneously or in successive steps as predetermined by definitive algorithms [34]. Abandoning the so-called batch analysis sessions in favour of a random access method firmly contributes to shifting the focus of the laboratory autoimmunologist from planning and executing the analytical procedure for each individual test to planning the diagnostic query appropriate for each individual patient as implied by the autoantibody test request [35].

Generally, the software that runs CLIA instruments destined for autoantibody assay includes algorithms for further investigations that the laboratory autoimmunologist can activate in “reflex” mode [36]. Thus, the patient’s entire laboratory diagnostic workup could be thought out and completed in a decidedly reduced timeframe, using a single serum sample. To that end, data obtained by Bentow and his team from 1079 consecutive clinical rheumatology patients demonstrated that the CLIA technology evaluated is adequate in terms of diagnostic sensitivity and specificity for the rapid screening (30 min) of a panel of specific anti-intracellular antigen antibodies (ENA) [37]. The same study emphasises that it is possible to simultaneously identify autoantibodies directed against all the most common intracellular antigens through the automatic reprocessing of only the positive ENA samples in order to evaluate the reactivity of the antigen involved.

## Turnaround time and effectiveness of response

It has already been demonstrated that automated CLIA, FEIA or Luminex analytic instrumentations permit the simultaneous detection of various single autoantibodies, achieving quantitative results in under an hour. Having such rapid access to autoantibody test results could be crucial for modern clinical practice considering the emergence of potentially life-threatening autoimmune pathologies that could lead to rapid progressive loss of vital organ function. These types of situations require a swift response to allow for a timely diagnosis and prompt initiation of efficacious immunosuppressive therapy [38–40].

This is the case, for example, with ANCA-associated small-vessel vasculitis, particularly Goodpasture syndrome with its characteristic circulating anti-glomerular basement membrane antibodies [41], and with Graves–Basedow disease, which is induced by anti-TSH receptor antibodies, in which thyroid crisis is the initial presentation [42, 43].

Another condition that requires prompt execution of specific antibody tests is a very rare complication called catastrophic antiphospholipid antibody syndrome [44]. For high-intensity clinical departments such as the intensive care unit, having fast access to autoantibody test results facilitates early diagnosis and the timely initiation of life-saving treatment.

## Changing operation protocols

Because of the intrinsic elevated diagnostic sensitivity and specificity associated with high productivity, introducing automated CLIA or FEIA methods into the autoimmunology laboratory is prompting the revision of the hierarchy of analytical methods currently in use for various diagnostic procedures.

For years, the Farr technique has represented the method of choice for anti-dsDNA detection and quantitative assay. Unfortunately, this technique has the disadvantage of utilising radioactive isotopes, thus requiring dedicated spaces for both test execution and waste disposal [45–48]. The indirect immunofluorescence (IIF) method, which uses Crithidia luciliae as substrate, is highly specific but not very sensitive: it is able to detect antibodies with high and intermediate avidity but is conditioned by operator subjectivity and is unsuitable for accurate quantitative determination. By contrast, immunoassay techniques are sensitive, are able to identify different immunoglobulin isotopes, are quantitative, can be automated and are not operator dependent. Furthermore, the current availability of novel antigenic preparations (recombinant, highly purified extracts, circular plasmids and synthetic oligonucleotides), along with other methodological improvements, has led to the production of new generation immunoassays that are able to detect anti-dsDNA only of intermediate and high avidity [49]. Consequently, the detection of anti-dsDNA autoantibodies for the diagnosis of SLE can be reliably executed by adopting the latest immunoassay techniques, which includes CLIA methods, and reports a quantitative result [50, 51].

Notwithstanding the recent progress gained with regards to analytical procedures and the recognition of fluoroscopic images by expert systems [52], the use of IIF methods for the detection of intracellular anti-antigen antibodies still presents certain methodological and interpretative limitations: they are time-consuming, poorly standardised and characterised by subjective interpretation and poor reproducibility of results. To overcome such limitations, numerous automated immunoassay methods have been developed (reviewed in [53]), which are generally characterised by superior productivity, greater reproducibility and easy of use, and lower operational costs as compared to IIF [54]. Nonetheless, the widely varying analytical and diagnostic performance of these techniques and non-correlation with results obtained by IIF [55, 56], prompted the American College of Rheumatology (ACR) in 2009 to reconfirm IIF as method of choice for the detection on anti-nuclear antibodies (ANA) [57]. In recent years, the progressive diffusion of immunochemical analysers utilised for the rapid assay of numerous autoantibodies in total automation, and the availability of new ANA screening tests that takes into account up to 16 different intracellular antigenic specificities bound to the solid phase, reopened the discussion on which analytic methodology to adopt for ANA detection. In this regard, a significant contribution has been made by a study that compared the diagnostic performance of a new test in chemiluminescence with that of the traditional IIF assay on a HEp-2 cellular substrate for ANA screening [58]. As could be predicted, in patients affected by autoimmune rheumatic disease the manual ANA IIF demonstrated greater sensitivity (81.5 vs. 78.1%) and lower specificity (79.4 vs. 94.1%) compared to CLIA. Surprising, however, was the difference in positivity between the CLIA (4.1%) and IIF (21.2%) tests in the control group, made up of healthy individuals, thus emphasising the clearly superior specificity of the CLIA assay. Even if per definition a screening test should be characterised by elevated sensitivity, it is similarly important that it should be adequately specific to avoid costly further investigations and/or unnecessary follow-up in healthy subjects. This final consideration is of particular importance when we bear in mind how the prescription of the co-called ANA tests has changed over the years. In the 1960s, when the IIF method was introduced, requesting it was the prerogative of but a few rheumatologists and immunologists; today, an ever-increasing number of physicians of vastly different specialities prescribe this test.

This rise in total number of requests as well as the increased number of inappropriate requests determines a drastic reduction in the pretest probability of disease and, as a result, calls for greater attention to the diagnostic specificity of the tests used for screening for anti-intracellular antigen autoantibodies [59].

ANA is not the only example of a possible application of the CLIA method. Historically, anti-neutrophil cytoplasmic antibodies (PR3 and MPO ANCA) have been analysed using the IIF method, with neutrophil granulocytes employed as substrate. Positive samples are confirmed by quantitative immunometric assay (ELISA, FEIA, etc.), which relies on purified human PR3 and MPO antigens bound to a solid phase. More recently, CLIA methods have been developed where the revelation of ANCA PR3 and MPO is based on capture or anchor systems that do not immobilise the antigens on solid phase by simple adsorption. These new methods, which guarantee optimal exposure of the conformational epitopes, showed analytical performance superior to other IIF methods [60–62]. The elevated sensitivity of the novel immunoassay methods, particularly that of the CLIA method, suggested that MPO and PR3 ANCA screening could be executed by using new analytical technologies [63, 64] and that results be confirmed by IIF [65]. However, even though numerous comparative studies have already demonstrated that CLIA boasts greater diagnostic accuracy, widespread consensus is yet to be reached on the best strategy to adopt for MPO and PR3 ANCA revelation [66–68].

## Potential future scenarios for CLIA technology

Regardless of its current optimal analytical performance, CLIA technology is destined for further development. The new flow-injection chemiluminescent immunoassay (FI-CLIA) technology which is based on the fast injection of micro-bubbles into the reaction system with the aim of ensuring a more efficient reagent mixture and of reducing incubation times and increasing temperature control, is able to improve the immunoreaction kinetics and therefore significantly reduce analysis time [69, 70].

Current chemiluminescent immunoassay consists of discrete tests, i.e. measures one autoantibody at a time. However, the need is emerging for multiparametric tests that can identify all the components of a complex immunological picture in a single analytical step, efficiently and at reasonable cost. Use of the two-dimensional resolution for CL multiplex immunoassay could open doors for the setting up of multiparametric CLIA tests. The technique is based on a multichannel sampling strategy, in combination with the use of various enzyme labels. After a brief period of batch incubation, the free conjugated enzymes separate from those bound to immunocomplexes and captured by magnetic microparticles; at the same time, activation of the magnet leads to the arrest of the immunological reaction. Subsequently, application of the channel-resolved approach makes it possible to record signals coming from various parallel measuring channels and thus enables the sequential determination of multiple analytes [71, 72].

Multianalyte assay has also been realised with the zone-resolved strategy, a method that implements the array: different antigens are immobilised in different positions on the solid phase in such a way that the individual immunoreactions can occur simultaneously and be distinctly individuated by array detectors [73]. Use of specific CCD (charge-coupled device) equipment accelerated the development of chemiluminescent imaging techniques and is making CLIA array more convenient compared to those employing alternative labels.

Finally, applying ultrasensitive chemiluminescence magnetic nanoparticles immunoassay (CL-MBs-nano-immunoassay) technology further increases the analytic sensitivity of the CLIA method. In this non-competitive and direct-type immunoassay, where the solid phase is made up of magnetic beads, gold nanoparticles with double labelling are used. One label is a monoclonal antibody specific for the analyte and the other label is horseradish peroxidase, thus amplifying the luminescent signal derived from the immunoreaction and the associated enzymatic reaction in an exponential manner [74, 75].

In conclusion, the evolutionary process of the CLIA method is, in all likelihood, merely beginning. In the coming years, new and more efficient analytical methods based on the principle of chemiluminescence will be introduced into autoimmune diagnostics, at steadily reduced cost. This transformation will align antibody diagnostics with already consolidated biochemistry and immunoassay methods, with noticeable advantages in terms of both diagnostic accuracy and expediency, to great clinical benefit.
